# Supplementary figures and images for: Molecular Mechanism Underlying the Sorghum sudanense (Piper) Stapf. Response to Osmotic Stress Determined via Single-Molecule Real-Time Sequencing and Next-Generation Sequencing
Source: Plants (Basel). 2023 Jul 12;12(14):2624. doi: 10.3390/plants12142624 (PMC10385767; doi:10.3390/plants12142624)

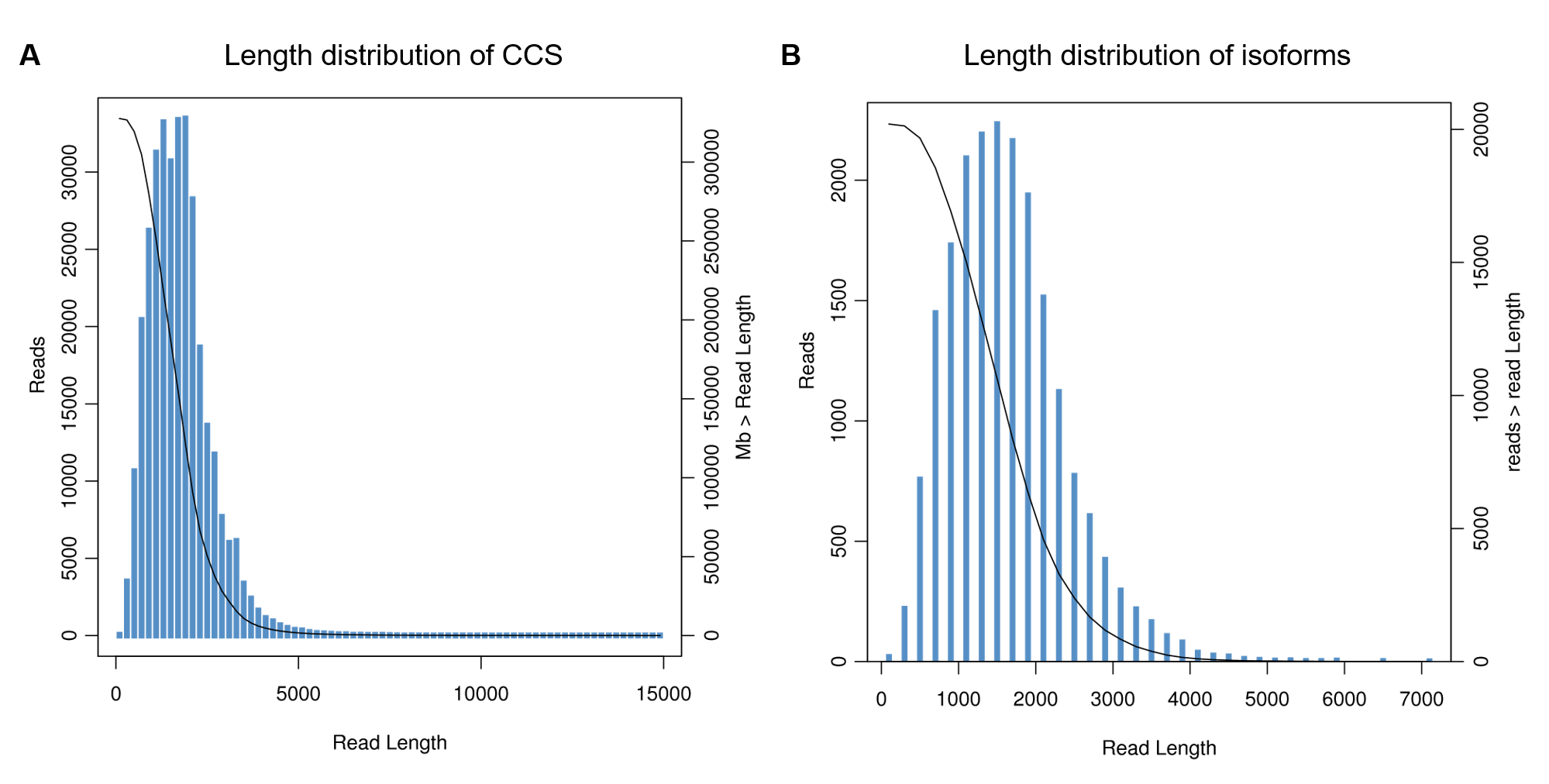

Supplement: Supplementary file 1 [file plants-12-02624-s001.zip › Figure S1.tif]

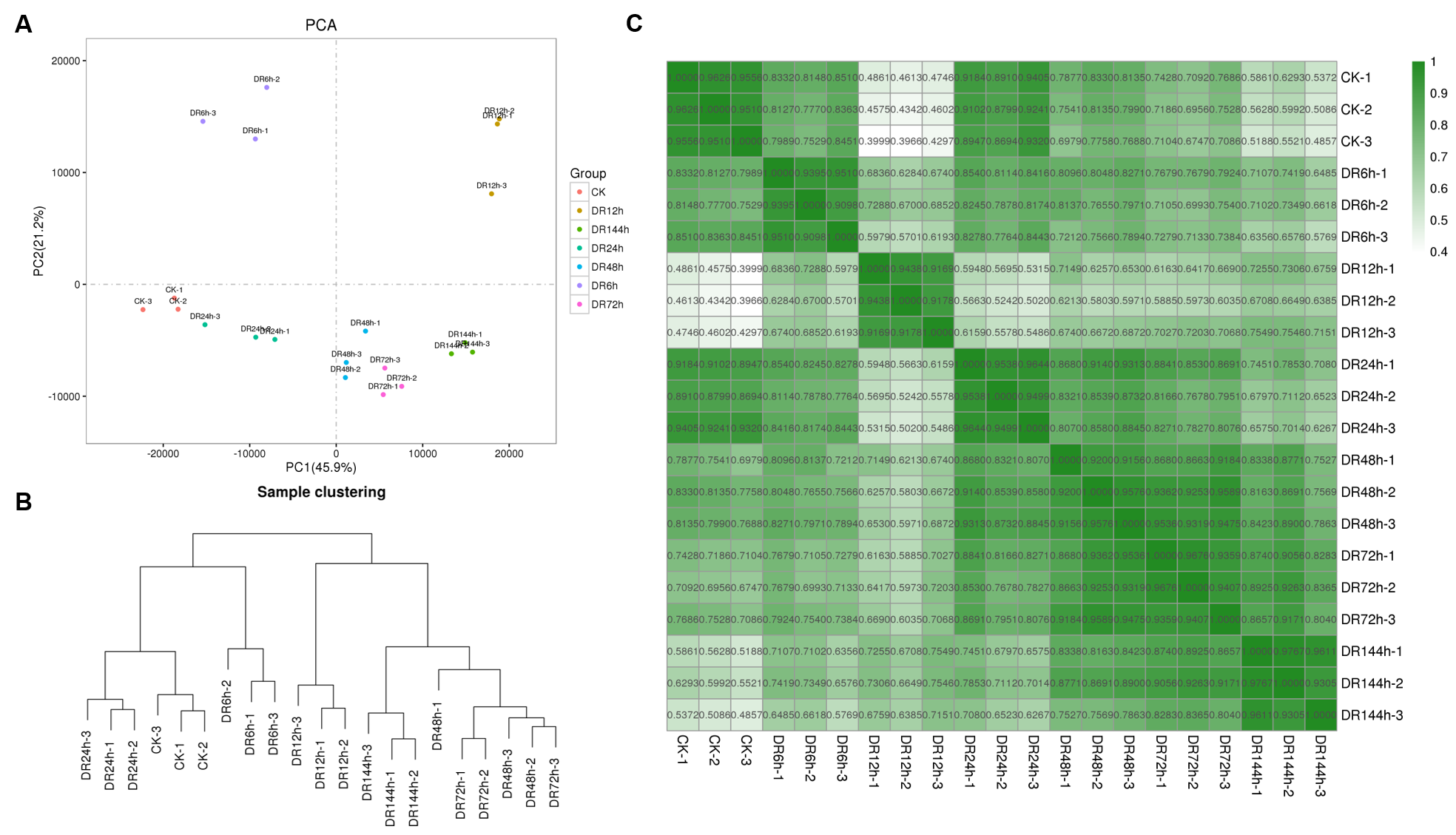

Supplement: Supplementary file 1 [file plants-12-02624-s001.zip › Figure S2.tif]
